# The Effects of Renal Denervation on Renal Hemodynamics and Renal Vasculature in a Porcine Model

**DOI:** 10.1371/journal.pone.0141609

**Published:** 2015-11-20

**Authors:** Willemien L. Verloop, Lisette E. G. Hubens, Wilko Spiering, Pieter A. Doevendans, Roel Goldschmeding, Ronald L. A. W. Bleys, Michiel Voskuil

**Affiliations:** 1 Department of Cardiology, University Medical Center Utrecht, Utrecht, the Netherlands; 2 Department of Vascular Medicine, University Medical Center Utrecht, Utrecht, the Netherlands; 3 Department of Pathology, University Medical Center Utrecht, Utrecht, the Netherlands; 4 Department of Anatomy, University Medical Center Utrecht, Utrecht, the Netherlands; University of Palermo, ITALY

## Abstract

**Rationale:**

Recently, the efficacy of renal denervation (RDN) has been debated. It is discussed whether RDN is able to adequately target the renal nerves.

**Objective:**

We aimed to investigate how effective RDN was by means of functional hemodynamic measurements and nerve damage on histology.

**Methods and Results:**

We performed hemodynamic measurements in both renal arteries of healthy pigs using a Doppler flow and pressure wire. Subsequently unilateral denervation was performed, followed by repeated bilateral hemodynamic measurements. Pigs were terminated directly after RDN or were followed for 3 weeks or 3 months after the procedure. After termination, both treated and control arteries were prepared for histology to evaluate vascular damage and nerve damage. Directly after RDN, resting renal blood flow tended to increase by 29±67% (P = 0.01). In contrast, renal resistance reserve increased from 1.74 (1.28) to 1.88 (1.17) (P = 0.02) during follow-up. Vascular histopathology showed that most nerves around the treated arteries were located outside the lesion areas (8±7 out of 55±25 (14%) nerves per pig were observed within a lesion area). Subsequently, a correlation was noted between a more impaired adventitia and a reduction in renal resistance reserve (β: -0.33; P = 0.05) at three weeks of follow-up.

**Conclusion:**

Only a small minority of renal nerves was targeted after RDN. Furthermore, more severe adventitial damage was related to a reduction in renal resistance in the treated arteries at follow-up. These hemodynamic and histological observations may indicate that RDN did not sufficiently target the renal nerves. Potentially, this may explain the significant spread in the response after RDN.

## Introduction

Chronic (hyper) activation of the sympathetic nervous system (SNS) is causative in the pathophysiology of hypertension.[1] Renal sympathetic nerves are part of this hyperactive system.[2] To investigate whether disruption of the renal nerves is effective in the treatment of hypertension, surgical renal denervation (RDN) has been studied upon. RDN appeared effective in preclinical studies and also in clinical studies that performed non-selective procedures such as radical surgical sympathectomy.[3, 4] Recently, a percutaneous approach was developed using radiofrequency (RF) energy to target the renal nerves.[3] The initial clinical studies were very promising.[5–7]. However, the only sham controlled randomized Symplicity HTN-3 trial showed disappointing results.[8] Moreover, a recent case-report demonstrated that the RF-energy induced damage to and around the vessel wall with limited penetration, leaving a large part of the nerves unaffected.[9] In this light, the aim of the current study was to investigate the level of renal nerve damage after RDN using histological techniques in a porcine model. The Symplicity Flex device (Medtronic, Minneapolis, USA) was used in the present study. Next to histological changes, the effect of RDN may also be measured by functional tests using hemodynamic measurements. Beforehand, we hypothesized that a reduction of resistance of the microvascular bed following RDN may lead to an improved renal blood flow. This improved renal perfusion may consequently reduce blood pressure (BP) in a clinical setting. To investigate this topic, intravascular hemodynamic measurements were performed before and directly after RDN.

## Methods

### Animals and study design

Thirteen female Dalland Landrace pigs (weight, 60–75 kg) received care in accordance with the *Guide for the Care and Use of Laboratory Pigs* prepared by the Institute of Laboratory Animal Resources. Experiments were approved by the Animal Experimentation Committee of the Medicine Faculty of the University of Utrecht, the Netherlands.

We intubated and anesthetized the animals using standard procedures. Using a sheath introducer in the femoral artery, a 6Fr RDC or IMA guiding catheter was introduced in the renal arteries for selective angiography. Hereafter, we performed hemodynamic measurements in both renal arteries. Subsequently, we treated one renal artery by RDN, followed by repeated hemodynamic measurements in both renal arteries. We terminated the first group of pigs (n = 3) directly after renal denervation. We terminated a second group of pigs (n = 5) 3 weeks after RDN and we terminated a third group of pigs (n = 5) 3 months after treatment. After termination, we processed both the treated and control renal arteries for histology.

### Hemodynamic measurements

We performed hemodynamic measurements in 10 pigs, using a 0,014-inch Combowire (Volcano Corporation, San Diego, CA, USA). This wire has a Doppler crystal located on the distal tip and a pressure sensor 3 cm proximal to the tip.[10] Pressure and flow velocity signals, combined with aortic pressure and ECG signals were recorded using the ComboMap system (Volcano Corporation). We recorded velocity and pressure signals during baseline and hyperemic conditions. Hyperemia was induced by an intra-arterial bolus of 20 mg papaverine. We assessed the hemodynamic measurements in both renal arteries prior to renal denervation and directly thereafter. After the predefined follow-up period of 3 weeks (n = 5) or 3 months (n = 5), we repeated the pressure and flow velocity measurements.

The data sets of the hemodynamic measurements were analyzed using AMC Studymanager, a custom software package (written in Delphi version 6.0, Borland Software Corporation and Delphi Version 2010, Embarcadero, San Francisco, CA, USA).[10] We calculated the average peak velocity (APV, expressed in cm/s) as the mean of four beats at baseline conditions (bAPV) and the mean of three successive beats at maximal hyperemia (hAPV). Pressure and flow velocity derived parameters were: aortic pressure (Paorta), baseline microvascular resistance (BMR), hyperemic microvascular resistance (HMR), renal flow velocity reserve (RFVR), and renal resistance reserve (RRR). The definitions of the evaluated parameters are shown in **Table 1**.

**Table 1:** Definitions of the parameters used.

Baseline microvascular resistance (BMR)	Paorta / bAPV
Hyperemic microvascular resistance (HMR)	Paorta / hAPV
Renal flow velocity reserve (RFVR)	hAPV / bAPV
Renal resistance reserve (RRR)	HMR / BMR

APV indicates average peak flow velocity; Paorta, aortic pressure; b, baseline; h, hyperemia

### Renal Denervation

We used the Symplicity flex device (Medtronic, Minneapolis, USA) to perform RDN. We performed a unilateral treatment of the renal arteries with at least 6 treatment points according to the previous described protocol.[11] It was randomly chosen whether the left or right renal artery was denervated. The treatment points were made in a helically way with a minimum of 5 mm distance in between.[11] After the procedure a control angiography was performed. The non-treated renal artery served as control vessel.

### Preparation for histology

We isolated the renal arteries and created a 2 to 3 cm wide renal stump. The renal stumps were dehydrated in ascending concentrations of alcohol and embedded in paraffin. Transverse sections of the renal artery were made after every 5 mm from its origin up to just distal to the main bifurcation of the renal artery resulting in 3 to 5 sectioning levels, dependent on the vessel length. On each level 5μm serial sections were cut and per artery all sectioning levels were mounted on a single glass slide.

### Histological examination for arterial damage

The detailed methods description is given **in Appendix in S1 File**. In brief, we examined the Masson’s trichrome (MST) and Haematoxylin Eosin (HE) stained sections for the location, type, and extent of vascular damage, neural damage and inflammation. Damage to the vessel wall within the lesional area and inflammation were assessed using scoring systems listed in the supplemental data **(Tables B and C in S1 File)**.[12, 13] Lesions were defined as an area of the vessel wall in which vascular integrity is disturbed, that is, a difference in staining within a vessel which is sharply set apart. The lesion is divergent in shape and extends from the intima to the adventitia direction. In all the different used antibodies, these differences between healthy and affected tissue were visible. For every antibody we used two 5μm sections. Per lesions this resulted in twelve 5μm sections. To determine whether loss of myofibroblasts was part of the vascular damage induced by RDN, we stained the slides with an antibody directed against alpha smooth muscle actin (alpha-SMA, **Appendix and Table A in S1 File)** and compared treated arteries with control arteries.

### Histological examination of nerve damage

After pretreatment the sections were immunohistochemically stained for the general neural marker protein gene product 9.5 (PGP9.5), the glial marker S-100, and for tyrosine hydroxylase (TH) which are markers for sympathetic nerve fibers. In the supplemental data detailed information about the used antibodies is given.

We examined the MST and HE stained sections for signs of neural degeneration such as pyknotic Schwann cells, digestion chambers, inflammation and/or peri-/endoneurial fibrosis.[14] We examined sections immunostained for PGP 9.5 for total nerve number. We examined sections immunostained for S-100 for the evaluation of Schwann cells within nerve fascicles. The intensity and distribution of TH staining was semi-quantitatively determined using a scoring system **(Table D in S1 File)**.[14]

### Statistical analysis

The variables about the hemodynamic measurements were reported as median (range), or as proportion when appropriate. We used the Wilcoxon signed rank test for paired sample analyses and the Mann-Whitney U test for non-paired sample analyses. The relation between change in hemodynamics (dependent variable) and histological parameters (independent variables) were analyzed using linear regression models. A two sided *P* value of <0.05 was considered statistically significant. All analyses were performed with the SPSS statistical package version 20 (IBM SPSS Data Collection, Chicago, IL).

## Results

All pigs had a renal anatomy eligible for treatment, particularly having a diameter of at least 4mm and a vessel length of at least 20mm.[15] All pigs were devoid of accessory renal arteries. All 13 pigs were treated unilaterally with 6–7 ablations per pig. Three pigs were terminated directly after RDN. The remaining 10 pigs were alive and healthy after a follow-up of respectively 3 weeks (n = 5) and 3 months (n = 5). Directly after RDN, we observed no macroscopic vascular complications like dissection or significant stenosis on angiograms, nor did we observe such complications at angiography during follow-up.

### Renal hemodynamic changes

Hemodynamic measurements were successful in 8 animals at follow-up; in 2 animals we were not able to capture an adequate signal for the measurements. **Table 2** presents the hemodynamic changes directly after RDN and at follow-up (combination of both 3 weeks and 3 months follow-up, no significant differences were observed between a follow-up of 3 weeks or 3 months). **S1 Fig** displays the hemodynamic measurements on screen. Directly after RDN resting average peak flow velocity (bAPV) increased by 29±67% (P = 0.01) and hyperemic APV (hAPV) increased by 39±54% (P = 0.04). The increase in APV was accompanied by a numerical increase in aortic pressure **(Table 2)**. Baseline microvascular resistance (BMR) and hyperemic resistance (HMR) did not significantly decrease by 12±35% (P = 0.18) and 14±28% (P = 0.13), respectively. Renal flow velocity reserve (RFVR) and renal resistance reserve (RRR) did also not chance directly after RDN.

**Table 2:** Overall change in renal hemodynamic parameters directly after renal denervation and after termination.

Hemodynamic parameters	Change directly after RDN			Change at termination (combination 3 weeks and 3 months)		
	Baseline	After RDN	P-value*	Baseline	Termination	P-value‡
**Aortic pressure**, mmHg	86 ± 18	92 ± 19	0.26	86 ± 18	87 ± 22	0.70
**Resting heart rate**, bpm	72 ± 22	72 ± 13	0.99	72 ± 22	70 ± 9	0.80
**Resting APV**, cm/sec	25 (19)	30 (43)	0.01	25 (19)	22 (19)	0.35
**Hyperemic APV**, cm/sec	43 (61)	47 (66)	0.04	43 (61)	39 (39)	0.61
**Resting MR**, mmHg/cm/sec	3.5 (6.8)	3.2 (3.5)	0.18	3.5 (6.8)	3.9 (4.7)	0.44
**Hyperemic MR**, mmHg/cm/sec	2.0 (0.8)	1.9 (1.5)	0.13	2.0 (0.8)	2.2 (2.4)	0.92
**Renal flow velocity reserve**	1.7 (1.3)	1.7 (1.6)	0.38	1.7 (1.3)	1.9 (1.1)	0.23
**Renal resistance reserve**	1.75 (1.61)	1.70 (2.08)	0.72	1.75 (1.61)	1.84 (1.34)	0.12

Continuous variables are displayed as mean ± SD or median (range). The Students T-test or Wilcoxon signed rank test were used for paired samples analysis. APV indicates average peak flow velocity, MR indicates microvascular resistance.

*Directly after measures compared to baseline measures,

‡Termination measures compared to baseline measures.

During follow-up, RRR increased in the treated arteries (mean increase compared to baseline 0.18±0.16; P = 0.02). This was accompanied by a trend towards increase in BMR (P = 0.09). Other indices at follow-up were not significantly different from baseline measurements in both the treated and control arteries.

Both before and directly after RDN, no significant differences in hemodynamic variables were observed between the treated and control arteries.

### Arterial damage

**Fig 1:**
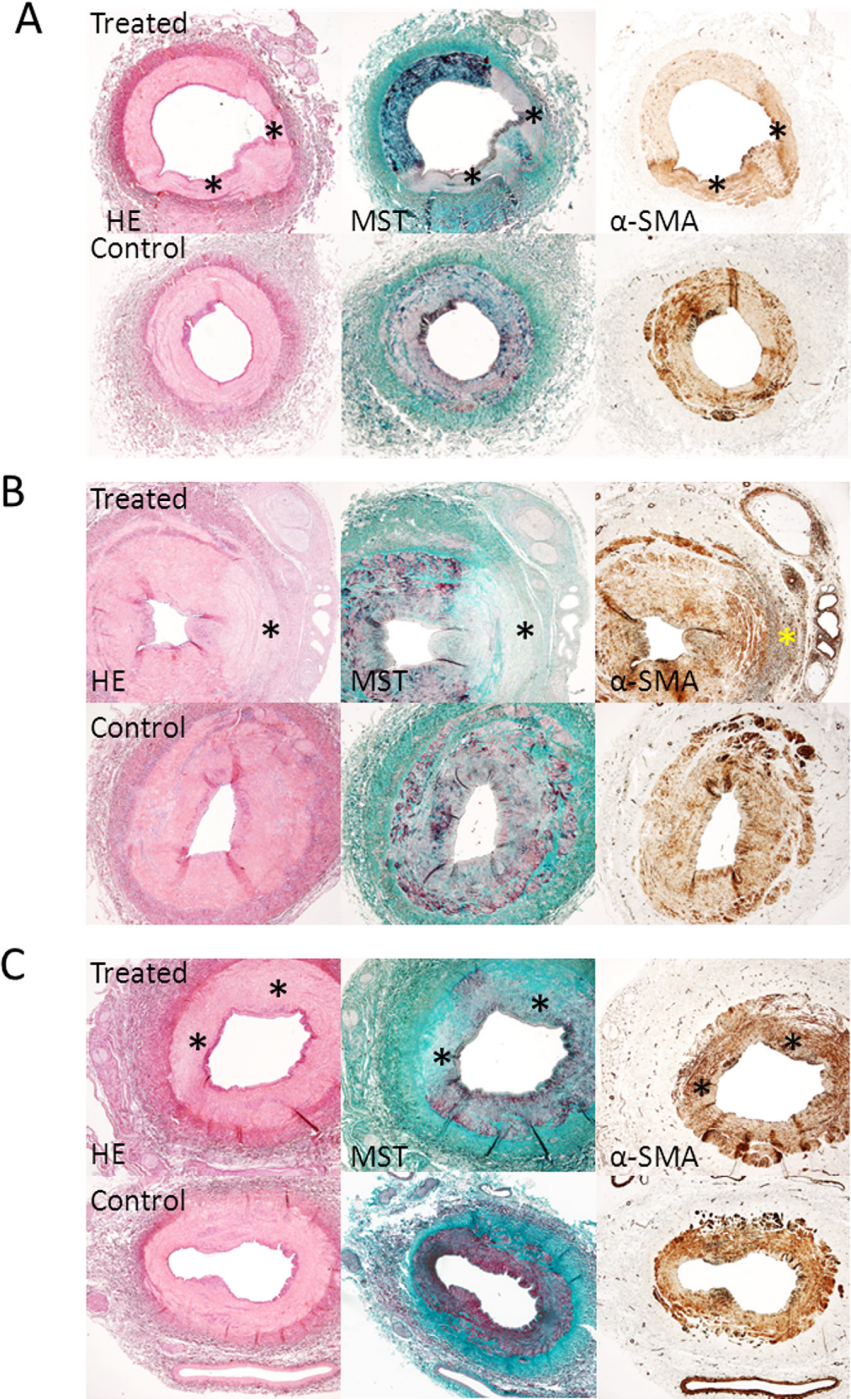
Acute, 3 weeks, and 3 months follow-up histology results. Acute (A), 3 weeks (B) and 3 months (C) histology results (2x magnification) showing treated vessels with lesions and control vessels. Serial sections were stained with Haematoxylin Eosin (HE), Masson’s trichome staining (MST) and alpha-smooth muscle actin (α-SMA immunostaining). * = lesion area. (A). HE and MST staining showing a treated vessel with two lesions immediately after denervation. The lesions have a pale color and the media is most affected. MST shows no increased collagen deposition at the site of the lesion (no increased presence of blue fibers). α-SMA staining shows a diffuse increased medial staining at the site of the lesion in treated vessels. (B) HE and MST staining at three weeks follow-up. The media and adventitia of treated vessels are most affected by denervation. MST staining shows increased medial collagen deposition (blue fibers). α-SMA staining shows increased medial, adventitial and perineural staining at the site of the lesion (dark brown). (C) HE and MST staining showing a treated vessel with two lesions 3 months after denervation. MST staining shows transmural collagen deposition at the site of the lesion and the adventitia is most affected. α-SMA staining shows a slightly increased medial staining (dark brown) at the site of the lesion.

**Fig 2:**
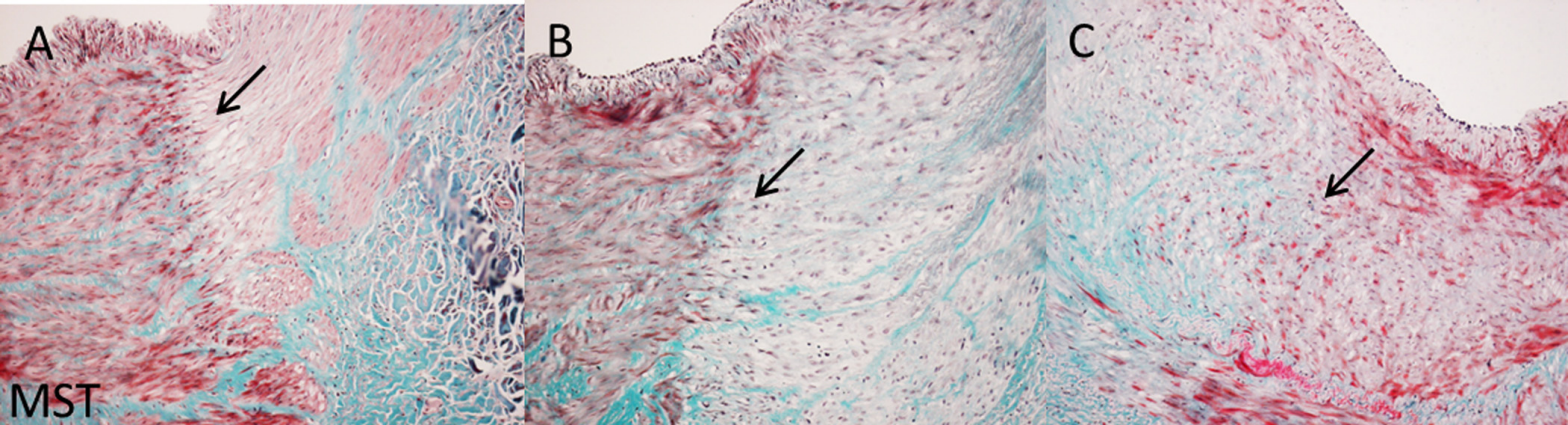
Tenfold magnification of Masson’s trichome staining of acute, 3 weeks, and 3 months follow-up histology. Acute (A), 3 weeks (B), and 3 months (C) histology results (10x magnification) showing the border zone of lesions in treated vessels. Sections were stained with MST staining. The arrows indicate the border zone. (A) Border zone of lesion immediately after denervation. The border zone is characterized by a red to pink color transition. At the border zone cell depletion is present (white holes). (B) Border zone of lesion three weeks after denervation. The border zone is characterized by a red to light green color transition. At the site of de border zone inflammatory cells are present (tiny dark blue/black spots). (C) Border zone of lesion 3 months after denervation. The border zone is characterized by blue to red color transition. At the border zone increased collagen deposition is present (blue fibers) and the collagen fibers are intertwined the adjacent healthy muscle tissue of the media (red).

**Fig 3:**
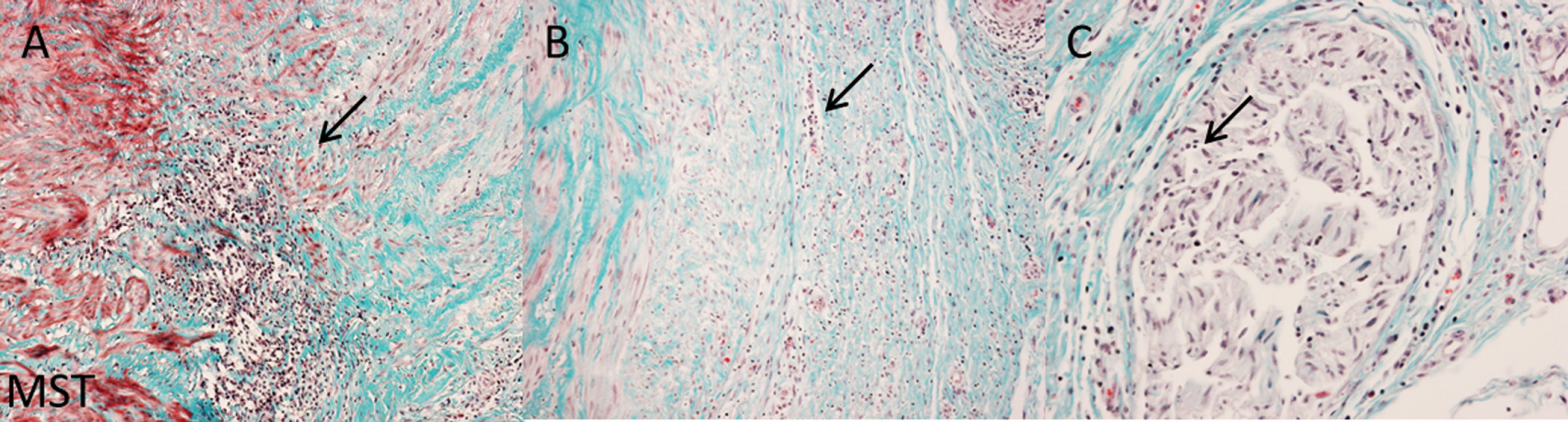
Extensive inflammatory response after 3 weeks follow-up. 3 week follow-up histology showing details of the extensive inflammatory response in lesions of treated vessels. Sections were stained with MST staining. Arrows indicate inflammatory cells. Inflammatory cells are tiny dark blue/ black spots. (A) A 10x magnification showing an extensive inflammatory response of the border zone at the media to adventitia transition. (B) A 10x magnification showing an extensive inflammatory response of the adventitia. (C) A 20x magnification showing inflammatory cells within a nerve.

**Fig 4:**
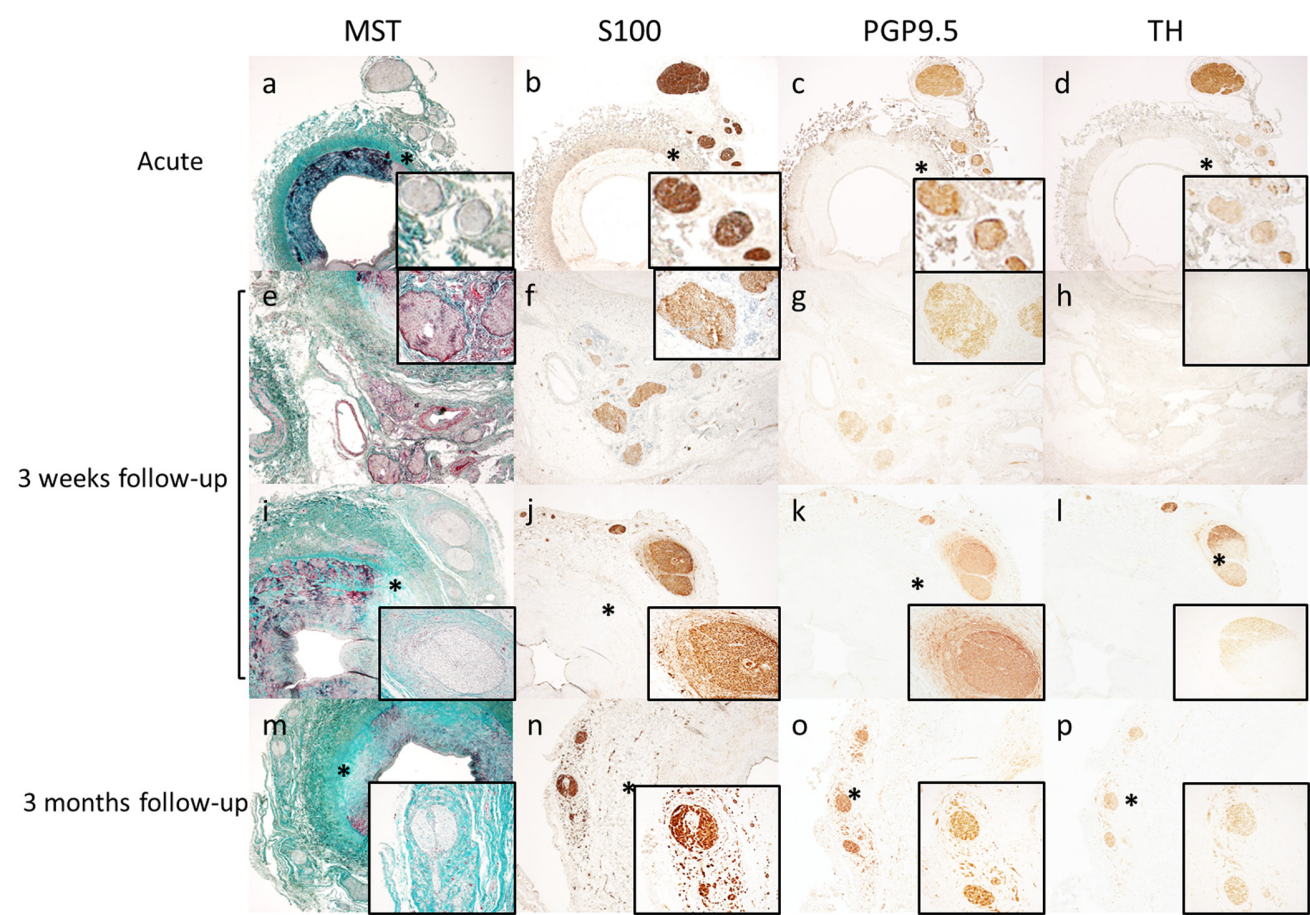
Immunohistochemical staining of nerves of treated arteries. Acute (A-D), 3 weeks (E-L) and 3 months (M-P) histology and immunohistochemical staining results of nerves within the lesion area of treated vessels. The rectangular boxes are a 10x magnification of the affected nerves. a-m shows a 2x magnification and n-p a 5x magnification. Immediately after denervation no signs of nerve damage and a scattered presence of inflammatory cells were observed (A). S100 (B), PGP9.5 (C), and TH (D) showed similar staining patterns and the staining intensity was similar to controls. 3 weeks after denervation neural degeneration and inflammation of nerves and perineural tissue (F) was observed. S100 staining intensity of affected nerves was similar to control (F,J) PGP9.5 staining was slightly lower in intensity (G,K)and TH (H,L)staining was weak or absent compared to control. Scattered presence of S100 (j)and PGP9.5 (K) positive neuron cell bodies was observed around a part of the affected nerves. 3 months after denervation the majority of nerves were embedded in thick sheets of fibrotic tissue(M) and there was scattered presence of inflammatory cells (M). S100, PGP9.5, and TH showed similar staining patterns and the staining intensity was similar to controls. Around affected nerves small S100 (N), PGP9.5 (O) and TH (P) positive nerve bundles were present and they were embedded in thick sheet of fibrotic tissue (M).

Histology was performed in eleven pigs. In two pigs histology failed due to technical issues. The geometrical changes are shown in Table 3, the vascular changes after RDN are shown in **Figs 1–4**. Directly after RDN we observed paralysis of the media, although this was non-significant between treated and control vessels (P = 0.11, **Table 3**). At three weeks, follow-up contraction of the media was observed, expressed as a trend towards a decreased ratio between circumference of the lumen and media (P = 0.08). At three months follow-up the ratio’s between the circumference of the lumen and the media were similar in the treated and control arteries (P = 0.66).

**Table 3:** Geometrical changes after renal denervation.

Moment	Diameter lumen (μm)			Ratio between the circumference of the lumen and the media					
	treated artery	control artery	P-value	treated artery	control artery	P-value	Percentage of intact media (%)	Diameter of intact media	Diameter of media within lesion
**Acute**	1459 (804)	841 (85)	0.11	0.70 (13)	0.51 (0.04)	0.11	57 (40)	509 (57)	270 (127)
**Three weeks**	1088 (736)	1143 (568)	0.23	0.50 (0.25)	0.55 (0.17)	0.08	67 (12.5)	574 (351)	695 (466)
**Three months**	1081 (618)	989 (784)	0.66	0.50 (0.18)	0.50 (0.19)	0.99	67 (25)	560 (198)	649 (301)

Ten out of eleven treated arteries that were evaluated showed clear vascular lesions **(Fig 2)**, one treated artery (follow-up duration 3 months) showed no vascular lesions. The RF-energy induced lesions up to 1,1 mm deep directly after RDN, up to 2,2 mm deep after 3 weeks follow-up, and up to 1,9 mm deep after 3 months follow-up. All lesions were in the form of a diverging spot. The supplemental data shows the grading of vascular damage **(Appendix and Table A in S2 File)**.

In the pigs that were terminated acutely, we observed in both treated and control arteries a scattered presence of inflammation cells. Immunostaining for alpha-SMA **(Fig 1A)** showed a diffuse and less intense staining within the lesions of the treated arteries compared to the control arteries.

At 3 weeks follow-up, we observed an extensive inflammation response in the treated arteries **(Fig 3)**, compared to a scattered presence in the control arteries. Immunostaining for alpha-SMA **(Fig 1B)** showed an increased staining within the lesion of treated arteries compared to control arteries. This suggests that in response to damage, myofibroblast proliferation is increased at the sites affected by RDN to heal the injured sites by deposition of collagen.

At 3 months follow-up, we observed scarring of the intima, media, and adventitia in two pigs **(Fig 1C)**. The media and adventitia could not be differentiated from each other since the external elastic lamina was no longer visible. Immunostaining for alpha-SMA **(Fig 1C)** showed slightly increased labeling in the media within the lesion of treated arteries compared to control arteries. In one pig we observed no clear vascular lesions three months after RDN.

### Immunohistochemical staining of nerve fibers

**Fig 5:**
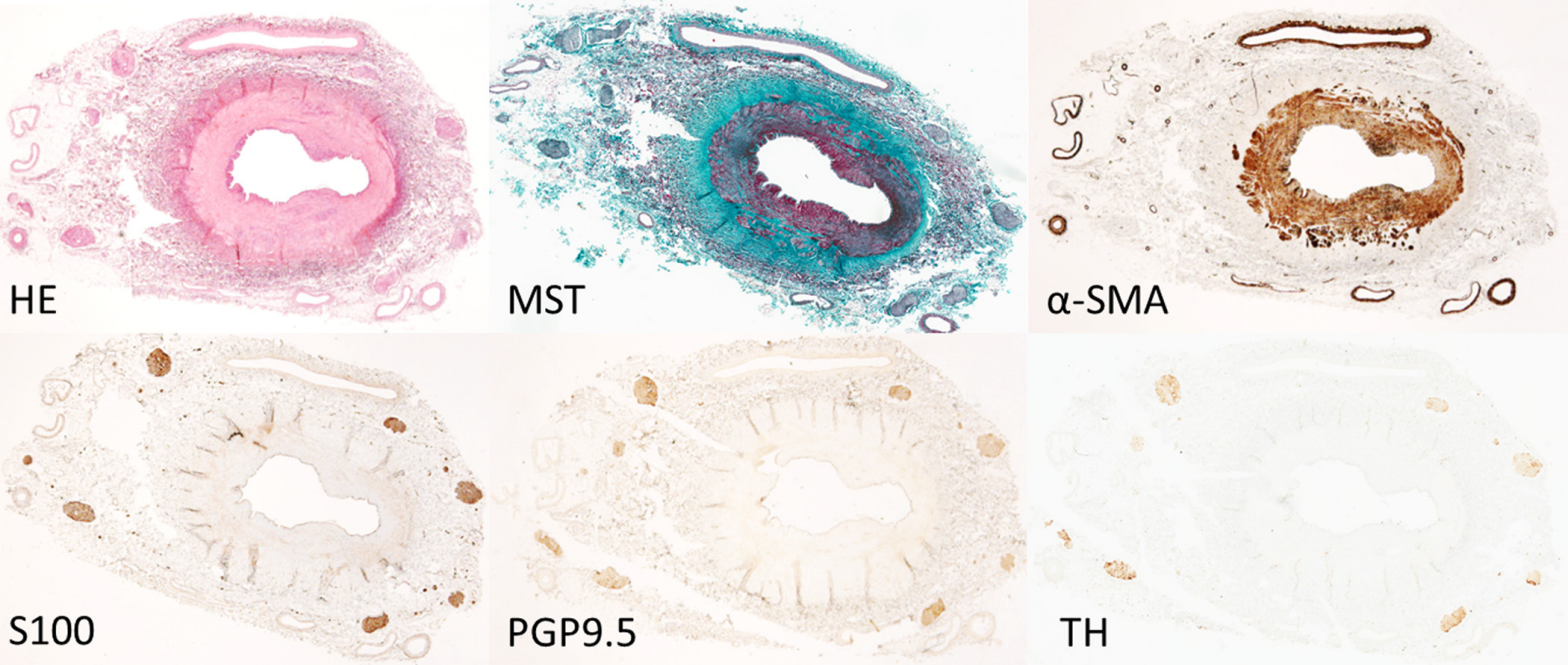
Histology and imunnostaining of control arteries. Histology results and immunostaining of a control vessel. Serial sections were stained for HE, MST, α-SMA, S100, PGP9.5 and TH. Sections show no signs of vessel damage (HE, MST), they have minimal inflammation (HE, MST) and have no increased areas of α-SMA at the media and no staining outside the media, except the arterioles. The nerves show similar staining patterns for structural (S100, PGP9.5) and functional (TH) nerve components.

**Fig 6:**
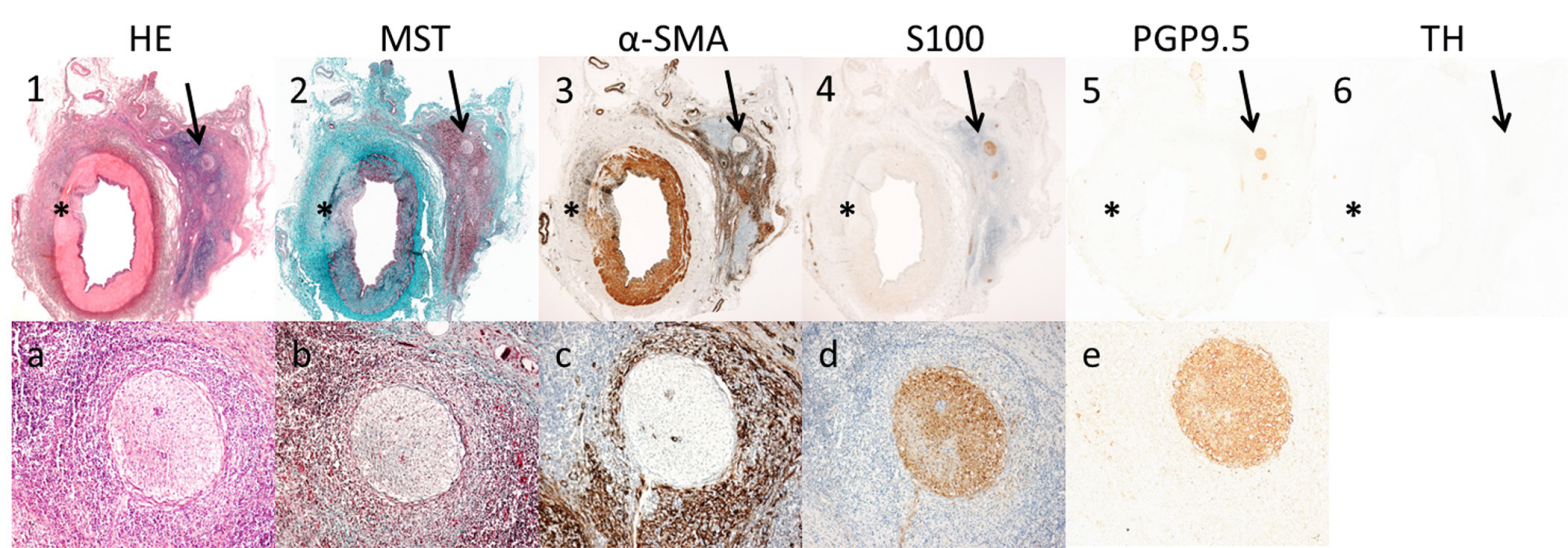
Nerve damage outside the lesion area. 3 weeks histology results showing a treated vessel with nerve damage outside the lesion area. A 20 x magnification (a-e) zooms in on the affected nerve that is indicated with an arrow in picture 1–6. Serial sections were stained with HE, MST, α-SMA, S100, PGP9.5 and TH. The perineurial tissue and nerves located at the opposite site of the lesion were affected by an extensive inflammatory response (1,A and 2,B), increased proliferation of myofibroblasts (3,C), a reduction in neural tissue (4,D;5,E) and loss of neurotransmitter production of the affected nerves (6).

**Figs 4–6** display the immunohistochemical staining of the nerve fibers. All nerves contained TH-positive nerve fibers **(Fig 5)**.

Directly after RDN, we observed no nerve damage with MST, PGP9.5, S100, and TH staining **(Figs 4 and 5)**. In the pigs with follow-up 55±25 nerves per pig were observed, only 8±7 of these nerves were within a lesion area **(Table B in S2 File)**. We analyzed the nerve fibers within the lesions area for possible damage.

After three weeks follow-up MST staining showed neural degeneration **(Figs 4 and 6)** of nerve fascicles with moderate to marked swelling of endoneurial tissue, proliferating Schwann cells, and severe inflammation **(Fig 4)**. PGP9.5 and S100 staining was slightly weaker in the nerves **(Fig 4)** of the treated arteries. TH staining intensity was weak or even absent in all nerves within a lesion **(Figs 4 and 6)**. This reduction of TH activity ranged from 10–90%.

After three months we only observed two bundles of nerve fibers in lesion areas **(Table B in S2 File)**. In these nerve fibers we observed peri-/endoneural fibrosis and inflammatory cells in the perineurium/epineurium **(Fig 4)**. Moreover, small PGP9.5, S100, and TH positive nerve fascicles were observed around a big nerve bundle in the treated arteries **(Fig 4)**. All other nerves bundles of treated arteries stained similar as control arteries, that showed normal morphology **(Fig 6)**.

### Relation between hemodynamic and histological findings

We observed a relation between a more impaired adventitia and a reduction in the renal resistance reserve (β: -0.33; P = 0.05) after three weeks follow-up in a univariate linear regression model.

## Discussion

The present study showed that RDN induced a variety of lesions in and around the arterial wall and a limited penetration up to 2 mm deep. Moreover, a large part of the renal nerves were remote to the vascular lumen and therefore not reached by the RF-energy. The observed nerve damage was presumably mediated by inflammation and it may be suggested that damaged nerves are able to recover, or that new axons grow. Moreover, we observed a perineural reaction outside the area of a vascular lesion. This might imply that damage to nerves is conducted. Whether this leads to reduced neural function is unknown. Directly after RDN we observed a trend towards an improvement in renal hemodynamics. However, most of the initial effects disappeared at follow-up. Remarkably, a reduction in RRR in the treated arteries at follow-up was related to more severe adventitial damage.

### Hemodynamic changes

This study was not the first evaluating hemodynamic changes after RDN in a porcine model. Yet, we could only partially confirm the results from the previously performed study.[16] In contrast to the previous study, we observed a nullification of most hemodynamic changes after follow-up.[16] This difference may be explained by the bilateral denervation in the previous study.

The increase in APV directly after RDN may be explained by the increase in aortic pressure since we did not observe a reduction in MR. At follow-up, we did observe an increase of the RRR in the treated arteries. Possible explanations for this increased RRR may be an ineffective RDN or reinnervation of the renal nerves.

Surprisingly, although, not statistically significant, we observed numerical changes in the hemodynamics in the control arteries, especially at follow-up. Potentially, RDN induced some changes in the kidneys itself, which consequently induced a systemic reflex from the afferent renal nerves. Such a systemic reflex would explain the hemodynamic changes on the contralateral side. Given the limited nerve damage, it is not unlikely that the hemodynamic response in the treated side is also explained by this systemic reflex through the efferent nerves of the treated side. However, all above is of course highly speculative. One should realize that we performed unilateral denervation and this may not be appropriate for assessment of hemodynamic changes after RDN. Ablation may interrupt the efferent en afferent fibers, but it does not seem to affect the reno-renal reflex. Therefore, the results about hemodynamic changes in the present study should be interpreted with caution. More important is the observation that only a limited number of fibers were affected.

### Histology

Regardless from follow-up duration and treatment or no treatment, we found that all nerves along the renal arteries contained many sympathetic fibers. This finding is in line with the available literature.[17]

Overall, the effects of RDN on the vasculature showed a wide variety and we observed lesions up to 2.2 mm deep, which is less deep than previously reported.[12] Moreover, one of the most surprising observations was that only a minority of the nerves (14%) around the treated arteries were captured within the vascular lesions. We could not find any explanation for this small number. This emphasizes the fact that RDN is a more or less “black box” procedure as the nerves cannot be visualized or mapped per procedurally yet.

Directly after RDN we observed no nerve damage in contrast to a previous study that showed reduced staining intensity of neurofilament protein and vacuolic appearance in a small number of nerve fascicles.[13] However, this may be explained by the fact that we observed a limited number of nerves within the lesions. We did observe minimal morphological changes in some nerves of the treated arteries (outside the lesion area). However, we also observed these changes in nerves of control arteries.

At three weeks follow-up the most prominent lesions were observed and the media, EEL, and adventitia were most affected by RDN. The massive inflammatory response represented most of the arterial and neural damage. Similar to Steigerwald et al., we observed neural inflammation, degeneration, and reduced marker expression at three weeks follow-up.[13] The weaker staining of PGP 9.5, S100, and TH may be explained by a loss of function from the affected nerves.

At three months follow-up, we only observed clear vascular damage in two out of three treated arteries. Since we only had a limited number or arteries with three months follow-up and vascular damage these results should be interpreted as explorative. In the arteries with vascular lesions, the EEL and adventitia were most affected by RDN. The inflammatory response had ceased and the entire lesion was scar tissue. Most nerves visualized where outside a vascular lesion area. Although the majority of the nerves had a thickened epi- and perineurium, the nerves were devoid of neural degeneration. These results were in accordance with the study of Rippy et al.[12] Although speculative, this may implicate that nerves are able to recover. In contrast, Henegar et al. showed that RDN can affect about 47% of the sympathetic fibers eight weeks after RDN[18]. As described above we observed a smaller number of affected nerves, mostly because most nerves were remote from the lesions. At present, we cannot explain the differences observed between the previous and present study. Potentially, it may be explained by the different catheters that were used.

### Limitations and strengths

Although we invested much effort to conduct the study as properly as possible, some limitations should be mentioned. Firstly, one can wonder whether current results in young healthy porcine arteries can be translated to RDN in renal arteries of patients with long-lasting (resistant) hypertension. It is likely that the arteries of hypertensive patients have an increased intima and/or media thickness and therefore show a different histopathologic pattern. Moreover, as we used a normotensive model the results about hemodynamic changes should be interpreted as a first step towards more investigation into hemodynamic changes. With the present model, we cannot draw any firm conclusions about the change in hemodynamics.

Secondly, we lack information about follow-up blood pressure and renal function. However, one would not expect BP effects in healthy pigs using RDN. Also, this was not the primary goal of the study. Finally, the number of pigs used for the current analysis was limited and most likely underpowered to detect any significant differences in hemodynamics between treated and control arteries.

Strengths of the current study were the comprehensive work-up and different follow-up durations used. The histological analyses of the vascular damage and nerve damage were extensive and were performed by an investigator that was blinded for the treatment site of RDN.

### Clinical implications

The current results may have some clinical implications. First of all, RDN resulted in only a limited penetration of the artery wall and the majority of the nerves around the arteries were not targeted by the RF-energy. It was recently shown that the mean distance from lumen to nerve is 3.12±0.54 mm in a human cadaver study.[19] The combination of the limited penetration and the location of nerves outside the lesion area may explain why a significant proportion of patients are ‘non-responder’ after RDN in ‘real-life’ cohorts.[20] Potentially, the absence of targeted nerves played a role in the negative HTN-3 trial.[8] Recently, Wang et al compared to different catheters: a saline-irrigated catheter (SIC) and a temperature-controlled catheter (TCC). [21] The findings of authors suggest that SIC ablation results in more extensive neural degeneration and deeper penetration.[21] Future research should point out if SIC ablation is more effective in a clinical setting to reduce blood pressure.

Surprising was the observation of the relation between changes in renal hemodynamics and histologically observed damage to the adventitia. This may implicate that deep lesions (e.g., up to the adventitia) are needed to establish these hemodynamic changes. In the development of new devices for RDN it should be kept in mind that deeper lesions may be needed to be more effective.

In conclusion, the present study demonstrated limited vascular and nerve damage after RDN.

The observations from the present study may indicate that the used RF catheter was not adequate to sufficiently target the renal nerves. The finding that a decreased RRR was related to more adventitial damage suggests that deeper vascular lesions are needed to induce hemodynamic improvements after RDN.

## Supporting Information

S1 Fig Hemodynamic measurements on screen. (TIF)

S1 File Appendix: methods extended. Table A: Antibodies. Table B: Histologic vascular injury grading scale. Table C: Inflammation of vascular and perivascular tissue: Grades 0–3. Table D: Tyrosine Hydroxylase staining: Grades 0–3.
(DOCX)

S2 File Appendix: results histology extended. Table A: Arterial damage. Table B: Number of damaged and total nerves relative to each lesion.
(DOCX)

S3 File Arrive checklist. (PDF)
