# Supplementary figures and images for: The Effects of Renal Denervation on Renal Hemodynamics and Renal Vasculature in a Porcine Model
Source: PLoS One. 2015 Nov 20;10(11):e0141609. doi: 10.1371/journal.pone.0141609 (PMC4654519; doi:10.1371/journal.pone.0141609)

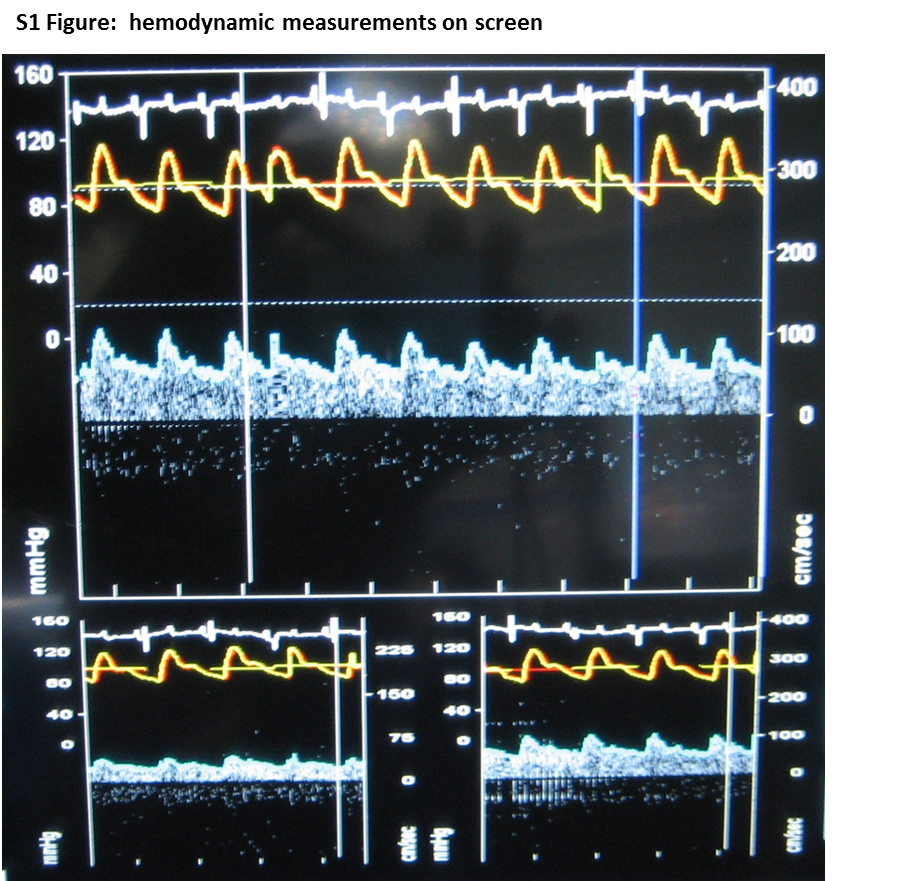

Supplement: S1 Fig — (TIF) [file pone.0141609.s001.tif]
