# Supplementary material for: The Effects of Renal Denervation on Renal Hemodynamics and Renal Vasculature in a Porcine Model
Source: PLoS One. 2015 Nov 20;10(11):e0141609. doi: 10.1371/journal.pone.0141609 (PMC4654519; doi:10.1371/journal.pone.0141609)
Supplement: S1 File — Table A: Antibodies. Table B: Histologic vascular injury grading scale. Table C: Inflammation of vascular and perivascular tissue: Grades 0–3. Table D: Tyrosine Hydroxylase staining: Grades 0–3. (DOCX) [file pone.0141609.s002.docx]

SUPPLEMENTAL MATERIAL

**S1 Appendix: methods extended**

**Histological staining protocols**

Prior to all stainings, the isolated renal stumps were embedded in paraffin. The renal stumps were sectioned every 5mm, producing 3 to 5 sectioning levels, depending on the length of the artery. Per artery all sections levels were captured on a single glass slide. The sections were cut approximately 5μm serially. All antibodies came from Sigma-Aldrich®, except the S-100 antibody that came from DAKO®. The specificity of the antibodies were tested within our laboratory with previous experiments with pigs.

*Mayer’s Haematoxilyn-Eosin staining*

Slides were deparaffinized and incubated with Mayer’s Haematoxylin for 15 minutes. Then slides were washed in running tap water for 15 minutes. Subsequently, slides were three times quickly rinsed in 80% ethanol. Then slides were incubated with eosin for 30 seconds, followed by differentiation in 96% ethanol. Slides were dehydrated with 100% ethanol (2 x 1 minute) and xylene (2 x 1 minute).

*Masson’s Trichrome staining*

Slides were deparaffinized and refixated in Bouin’s fluid for 1 hour at 56°C. The nuclei were stained with Weigert’s iron haematoxylin for 10 minutes. Then the slides were washed with running tapwater for 10 minutes and rinsed with distilled water. Subsequently, slides were incubated with solution A (0.5g Acid fuchsine, 0.5g Xylidine ponceau and 1 ml glacial acetic acid in 99ml distilled water) for 10 minutes and rinsed with distilled water. Then slides were incubated with solution B ( 1g phosphotungstic acid in 100 ml distilled water) for 5 minutes and rinsed with distilled water. Finally slides were incubated with solution C (2g light green SF yellowfish and 2ml glacial acetic acid in 100ml distilled water) for 10 minutes and dehydrated.

**Immunostaining protocols**

Slides were deparaffinized and pretreated using heat activated antigen retrieval in sodium-citrate buffer (0.01M, pH 6.0). Endogenous peroxidase activity was blocked using 3% hydrogen peroxidase in distilled water for 5 minutes. Slides were subsequently incubated with a protein block, primary and secondary antibody according to table 1. Detection of primary antibody binding for Alpha-SMA, PGP9.5, TH and S100 was performed by incubation with Sigma Fast DAB for 10 minutes.

**S1 Table A** Antibodies

| **Block** | **Primary antibody**  **(Species, dilution)** | **Incubation time primary antibody** | **Secondary antibody**  **(dilution)** | **Incubation time secondary antibody** |
| --- | --- | --- | --- | --- |
| None | Alpha-SMA (1:3200) | 32 minutes at RT | Ready-to-use poly HRP anti mouse/rabbit IgG | 16 minutes at RT |
| Ultra V | PGP9.5 (rabbit, 1:1000) | 2 hours at RT | Ready-to-use poly HRP anti mouse/rabbit/rat IgG | 30 minutes at RT |
| None | S100 (1:2000) | 32 minutes at RT | Ready-to-use poly HRP anti mouse/rabbit IgG | 16 minutes at RT |
| Ultra V | TH (rabbit, 1:400) | 2 hours at RT | Ready-to-use poly HRP anti mouse/rabbit/rat IgG | 30 minutes at RT |

**Histopathological grading scales**

**S1 Table B** Histologic vascular injury grading scale

*Injury and/or disruption of the intima (IH), internal elastic lamina (IEL), media, external elastic lamina (EEL), and adventitia: Grades 0-5*

| 0 | no injury/disruption/hyperplasia |
| --- | --- |
| 1 | minimal (injury/disruption hyperplasia of 10% or less) |
| 2 | mild (injury/disruption/hyperplasia of 11-25%) |
| 3 | moderate (injury/disruption/hyperplasia of 26-50%) |
| 4 | marked (injury/disruption/hyperplasia of 51-75%) |
| 5 | severe (injury/disruption/hyperplasia of 76-100%) |

**S1 Table C** Inflammation of vascular and perivascular tissue: Grades 0-3

| 0 | no inflammation |
| --- | --- |
| 1 | presence of scattered inflammation cells |
| 2 | modest inflammatory reaction comprising less than 25% of the vessel circumference |
| 3 | massive inflammatory reaction comprising more than 25% of the vessel circumference |

**S1 Table D** Tyrosine Hydroxylase staining: Grades 0-3

| 0 | no reaction |
| --- | --- |
| 1 | patchy/very weak reaction |
| 2 | weak reaction |
| 3 | strong reaction |
