# Supplementary material for: The Effects of Renal Denervation on Renal Hemodynamics and Renal Vasculature in a Porcine Model
Source: PLoS One. 2015 Nov 20;10(11):e0141609. doi: 10.1371/journal.pone.0141609 (PMC4654519; doi:10.1371/journal.pone.0141609)
Supplement: S2 File — Table A: Arterial damage. Table B: Number of damaged and total nerves relative to each lesion. (DOCX) [file pone.0141609.s003.docx]

**S2 Appendix: results histology extended**

**S2 table A.** Arterial damage

|  | **Acute termination** | **Three weeks follow-up** | **Three months follow-up** |
| --- | --- | --- | --- |
| **Intima** | Grade 3 | Grade 2 | Grade 1 |
| **Internal elastic lamina** | Grade 3 | Grade 2 | Grade 1 |
| **Media** | Grade 4 | Grade 3 | Grade 2 |
| **External elastic lamina** | Grade 4 | Grade 3 | Grade 3 |
| **Adventitia** | Grade 2 | Grade 3 | Grade 3 |
| **Inflammation** | Grade 1 | Grade 3 | Grade 1 |
| **Inflammation control artery** | Grade 1 | Grade 1 | Grade 1 |

Pigs that were terminated directly after RDN: Damage was observed as thrombus formation at the site of the lesion, stretched fibers of the IEL and EEL, retraction of the media, cell depletion at the borders of the lesion e.g. ‘border zone’ (figure 2), and coagulation of the adventitia. Immunostaining for alpha-SMA (figure 1) showed a diffuse and less intense staining within the lesions of the treated arteries compared to the control arteries.

At 3 weeks follow-up, the damage was observed as intimal hyperplasia, intimal and medial fibrosis, and adventitial inflammation. At the border zone invasion of leukocytes and deposition of collagen was present (figure 3, 4). Immunostaining for alpha-SMA (figure 1) showed an increased staining within the lesion of treated arteries compared to control arteries.

At 3 months follow-up, we observed vascular injury in one of the two treated arteries. Damage was observed as scarring of the intima, media, and adventitia. The media and adventitia could not be differentiated from each other since the external elastic lamina was no longer visible. The border zone was less pronounced because the collagen fibers of the scar tissue were intertwined with the adjacent healthy tissue (figure 3). Immunostaining for alpha-SMA (figure 2) showed a slightly increased labeling in the media within the lesion of treated arteries compared to control arteries.

*Immunohistochemical staining of nerve fibers*

All nerves contained TH-positive nerve fibers (figure 6).

Masson’s trichome staining in the pigs that were terminated directly showed no nerve damage. PGP9.5, S100, and TH staining showed similar intensity (grade 3) for treated (figure 6) and control arteries (figure 5).

Masson’s trichome staining of the treated arteries of the pigs with three weeks follow-up showed neural degeneration (figure 6) of nerve fascicles with moderate to marked swelling of endoneurial tissue, proliferating Schwann cells, and severe inflammation (figure 3). PGP9.5 and S100 staining was slightly weaker in the nerves (figure 6) of the treated arteries compared to the control arteries. TH staining intensity was weak or even absent in the treated arteries (grade 1, figure 6), whereas in the control arteries staining intensity was strong (grade 3, figure 5). A scattered presence of PGP9.5 and S100 positive neuron cell bodies was observed around damaged nerve fibers of the treated arteries (figure 6).

Masson’s trichome staining of the treated arteries of the pigs with three months follow-up showed peri-/endoneurial fibrosis and occasional inflammatory cells in the perineurium/epineurium (figure 6). The degree of neural degeneration was similar in the treated and control arteries. The majority of nerves of the treated arteries had a thickened perineurium. Immunostaining for TH (grade 2), PGP9.5, and S100 was similar for both treated (figure 6) and control arteries (figure 5). In the treated artery that contained vascular damage, small PGP9.5, S100, and TH positive nerve fascicles were observed around a big nerve bundle (figure 6). They were all surrounded by thick sheets of fibrotic epi- and perineurium.

**S2 Table B.** Number of damaged and total nerves relative to each lesion

|  | Nerve count within lesion section 1 | Nerve count within lesion section 2 | Nerve count within lesion section 3 | Nerve count within lesion section 4 | Nerve count within lesion section 5 | Damaged nerve count within lesion section 1 | Damaged nerve count within lesion section 2 | Damaged nerve count within lesion section 3 | Damaged nerve count within lesion section 4 | Damaged nerve count within lesion section 5 |
| --- | --- | --- | --- | --- | --- | --- | --- | --- | --- | --- |
| acute | 0 | 6 | 0 | 4 | 0 | 0 | 0 | 0 | 0 | 0 |
| acute | 0 | 0 | 0 | 0 | 0 | 0 | 0 | 0 | 0 | 0 |
| acute | 1 | 0 | 0 | 0 | 0 | 0 | 0 | 0 | 0 | 0 |
| 3 weeks | 0 | 1 | 14 | 0 | 0 | 0 | 1 | 10 | 0 | 0 |
| 3 weeks | 0 | 0 | 0 | 3 | 0 | 0 | 0 | 0 | 3 | 0 |
| 3 weeks | 0 | 0 | 7 | 8 | 0 | 0 | 0 | 5 | 7 | 0 |
| 3 weeks | 0 | 0 | 0 | 0 | 0 | 0 | 0 | 0 | 0 | 0 |
| 3 weeks | 0 | 0 | 0 | 0 | 0 | 0 | 0 | 0 | 0 | 0 |
| 3 months | 0 | 0 | 3 | 4 | 0 | 0 | 0 | 0 | 0 | 0 |
| 3 months | 0 | 0 | 0 | 0 | 0 | 0 | 0 | 0 | 0 | 0 |
| 3 months | 0 | 0 | 2 | 0 | 1 | 0 | 0 | 1 | 0 | 0 |
